# Supplementary material for: Genomic and Transcriptomic Characteristics According to Size of Papillary Thyroid Microcarcinoma
Source: Cancers (Basel). 2020 May 25;12(5):1345. doi: 10.3390/cancers12051345 (PMC7281223; doi:10.3390/cancers12051345)
Supplement: Supplementary file 1 [file cancers-12-01345-s001.pdf]

Supplementary Materials

Genomic and Transcriptomic Characteristics According to Size of Papillary Thyroid Microcarcinoma

Young Shin Song, Byung-Hee Kang, Seungbok Lee, Seong-Keun Yoo, Young Sik Choi, Jungsun Park, Dong Yoon Park, Kyu Eun Lee, Jeong-Sun Seo and Young Joo Park

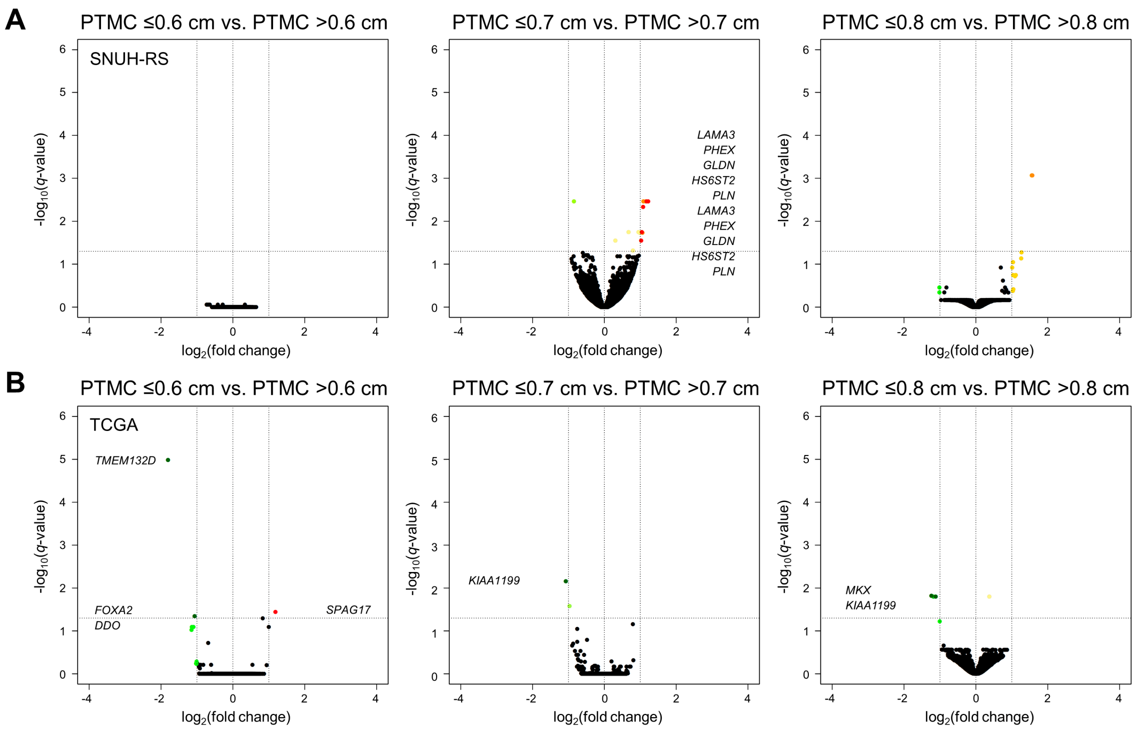

Figure S1. Transcriptomic characteristics of *BRAF*-mutant papillary thyroid microcarcinoma according to tumor size. Volcano plots showing differential RNA expression levels between groups of *BRAF*-mutant PTMCs: ≤0.6 cm vs. >0.6 cm (left), ≤0.7 cm vs. >0.7 cm (middle), and ≤0.8 cm vs. >0.8 cm (right) from the **(A)** SNUH RNA-sequencing (SNUH-RS, n = 11 vs.. 18, 18 vs. 11, 22 vs. 7, respectively) and **(B)** TCGA (n = 3 vs. 15, 6 vs. 12, 7 vs. 11, respectively) datasets. PTMC, papillary thyroid microcarcinoma.

Table S1. Information of datasets included in the study.

Dataset	No. of total PTCs	No. of PTMCs	Sequencing type	Information used for analysis
SNUH (1)	221 [a]	93 [a]	Targeted sequencing; Sanger sequencing	Mutations of 612 genes and *TERT* promoter
(2)	312 [b]	123 [b]	Sanger sequencing	Mutations of *BRAF*^{V600E}, *RAS*, and *TERT* promoter
(3)	2282 [b]	1687 [b]	Sanger sequencing	*BRAF*^{V600E} mutation
(4)	124	43	RNA sequencing; Sanger sequencing	Mutations of *BRAF*^{V600E}, *RAS*, and *TERT* promoter; Whole transcriptome
TCGA (5)	496	39	Whole exome/genome sequencing; Targeted sequencing; RNA sequencing	Mutations of whole genes and *TERT* promoter; Whole transcriptome
Total	3435	1985		

(1) New dataset of this study, using targeted sequencing and Sanger sequencing for *TERT* promoter mutations (represented as the SNUH targeted-sequencing dataset (SNUH-TS) in figures).

(2) Song, Y.S.; Lim, J.A.; Choi, H.; Won, J.K.; Moon, J.H.; Cho, S.W.; Lee, K.E.; Park, Y.J.; Yi, K.H.; Park, D.J., et al. Prognostic effects of *TERT* promoter mutations are enhanced by coexistence with *BRAF* or *RAS* mutations and strengthen the risk prediction by the ATA or TNM staging system in differentiated

thyroid cancer patients. *Cancer* **2016**, *122*, 1370-1379.
(3) Hong, A.R.; Lim, J.A.; Kim, T.H.; Choi, H.S.; Yoo, W.S.; Min, H.S.; Won, J.K.; Lee, K.E.; Jung, K.C.; Park, D.J., et al. The frequency and clinical implications of the *BRAF*(V600E) mutation in papillary thyroid cancer patients in Korea over the past two decades. *Endocrinol Metab (Seoul)* **2014**, *29*, 505-513.
(4) Yoo, S.K.; Lee, S.; Kim, S.J.; Jee, H.G.; Kim, B.A.; Cho, H.; Song, Y.S.; Cho, S.W.; Won, J.K.; Shin, J.Y., et al. Comprehensive analysis of the transcriptional and mutational landscape of follicular and papillary thyroid cancers. *PLoS Genet* **2016**, *12*, e1006239 (represented as the SNUH RNA-sequencing dataset (SNUH-RS) in figures).
(5) Cancer Genome Atlas Research Network. Integrated genomic characterization of papillary thyroid carcinoma. *Cell* **2014**, *159*, 676-690 (represented as the Cancer Genome Atlas dataset (TCGA) in figures).
[a] PTCs were selected if the residual DNA was available for additional sequencing analysis, among the PTCs in Datasets (2) and (3).
[b] The number of PTCs in Studies (2) and (3) is the number except when included in Dataset (1).

Table S2. List of genes included in the SNUH targeted-sequencing dataset.

**Kinase Genes (*n* = 566)**							
AAK1	*AATK*	*ABL1*	*ABL2*	*ACTR2*	*ACVR1*	*ACVR1B*	*ACVR1C*
ACVR2A	*ACVR2B*	*ACVRL1*	*ADCK1*	*ADCK4*	*ADCK5*	*ADRBK1*	*ADRBK2*
AGK	*AKT1*	*AKT2*	*AKT3*	*ALK*	*ALPK1*	*ALPK2*	*ALPK3*
AMHR2	*ANKK1*	*ARAF*	*ATM*	*ATR*	*AURKA*	*AURKB*	*AURKC*
AXL	*BCKDK*	*BLK*	*BMP2K*	*BMPR1A*	*BMPR1B*	*BMPR2*	*BMX*
BRAF	*BRD2*	*BRD3*	*BRD4*	*BRDT*	*BRSK1*	*BRSK2*	*BTK*
BUB1	*BUB1B*	*C9orf96*	*CABC1*	*CAMK1*	*CAMK1D*	*CAMK1G*	*CAMK2A*
CAMK2B	*CAMK2D*	*CAMK2G*	*CAMK4*	*CAMKK1*	*CAMKK2*	*CAMKV*	*CASK*
CCRK	*CDC2*	*CDC2L2*	*CDC2L5*	*CDC2L6*	*CDC42BPA*	*CDC42BPB*	*CDC42BPG*
CDC7	*CDK10*	*CDK2*	*CDK3*	*CDK4*	*CDK5*	*CDK6*	*CDK7*
CDK8	*CDK9*	*CDKL1*	*CDKL2*	*CDKL3*	*CDKL4*	*CDKL5*	*CERK*
CHEK1	*CHEK2*	*CHUK*	*CIT*	*CLK1*	*CLK2*	*CLK3*	*CLK4*
CNKSR2	*CRKRS*	*CSF1R*	*CSK*	*CSNK1A1*	*CSNK1A1L*	*CSNK1D*	*CSNK1E*
CSNK1G1	*CSNK1G2*	*CSNK1G3*	*CSNK2A1*	*CSNK2A2*	*DAPK1*	*DAPK2*	*DAPK3*
DCLK1	*DCLK2*	*DCLK3*	*DDR1*	*DDR2*	*DGKA*	*DGKB*	*DGKD*
DGKE	*DGKG*	*DGKH*	*DGKI*	*DGKQ*	*DGKZ*	*DMPK*	*DSTYK*
DYRK1A	*DYRK1B*	*DYRK2*	*DYRK3*	*DYRK4*	*EEF2K*	*EGFR*	*EIF2AK1*
EIF2AK2	*EIF2AK3*	*EIF2AK4*	*EPHA1*	*EPHA10*	*EPHA2*	*EPHA3*	*EPHA4*
EPHA5	*EPHA6*	*EPHA7*	*EPHA8*	*EPHB1*	*EPHB2*	*EPHB3*	*EPHB4*
EPHB6	*ERBB2*	*ERBB3*	*ERBB4*	*ERN1*	*ERN2*	*FASTK*	*FER*
FES	*FGFR1*	*FGFR2*	*FGFR3*	*FGFR4*	*FGR*	*FLJ25006*	*FLT1*
FLT3	*FLT4*	*FRK*	*FYN*	*GAK*	*GCK*	*GRK1*	*GRK4*
GRK5	*GRK6*	*GRK7*	*GSG2*	*GSK3A*	*GSK3B*	*GUCY2C*	*GUCY2D*
GUCY2F	*HCK*	*HIPK1*	*HIPK2*	*HIPK3*	*HIPK4*	*HSPB8*	*HUNK*
ICK	*IGF1R*	*IKBKB*	*IKBKE*	*ILK*	*INSR*	*INSRR*	*IP6K1*
IP6K2	*IP6K3*	*IPMK*	*IPPK*	*IRAK1*	*IRAK2*	*IRAK3*	*IRAK4*
ITK	*ITPK1*	*ITPKA*	*ITPKB*	*ITPKC*	*JAK1*	*JAK2*	*JAK3*
KALRN	*KDR*	*KIAA1804*	*KIT*	*KSR1*	*KSR2*	*LATS1*	*LATS2*
LCK	*LIMK1*	*LIMK2*	*LMTK2*	*LMTK3*	*LRRK1*	*LRRK2*	*LTK*
LYN	*MAK*	*MAP2K1*	*MAP2K2*	*MAP2K3*	*MAP2K4*	*MAP2K5*	*MAP2K6*
MAP2K7	*MAP3K1*	*MAP3K10*	*MAP3K11*	*MAP3K12*	*MAP3K13*	*MAP3K14*	*MAP3K15*
MAP3K2	*MAP3K3*	*MAP3K4*	*MAP3K5*	*MAP3K6*	*MAP3K7*	*MAP3K8*	*MAP3K9*
MAP4K1	*MAP4K2*	*MAP4K3*	*MAP4K4*	*MAP4K5*	*MAPK1*	*MAPK10*	*MAPK11*
MAPK12	*MAPK13*	*MAPK14*	*MAPK15*	*MAPK3*	*MAPK4*	*MAPK6*	*MAPK7*
MAPK8	*MAPK9*	*MAPKAPK2*	*MAPKAPK3*	*MAPKAPK5*	*MARK1*	*MARK2*	*MARK3*
MARK4	*MAST1*	*MAST2*	*MAST3*	*MAST4*	*MASTL*	*MATK*	*MELK*
MERTK	*MET*	*MGC42105*	*MINK1*	*MKNK1*	*MKNK2*	*MLKL*	*MOS*
MST1R	*MST4*	*MTOR*	*MUSK*	*MYLK*	*MYLK2*	*MYLK3*	*MYLK4*
MYO3A	*MYO3B*	*NEK1*	*NEK10*	*NEK11*	*NEK2*	*NEK3*	*NEK4*
NEK5	*NEK6*	*NEK7*	*NEK8*	*NEK9*	*NLK*	*NPR1*	*NPR2*
NRBP1	*NRBP2*	*NRK*	*NTRK1*	*NTRK2*	*NTRK3*	*NUAK1*	*NUAK2*

OBSCN	*OXSR1*	*PAK1*	*PAK2*	*PAK3*	*PAK4*	*PAK6*	*PAK7*
PASK	*PBK*	*PCTK1*	*PCTK2*	*PCTK3*	*PDGFRA*	*PDGFRB*	*PDIK1L*
PDK1	*PDK2*	*PDK3*	*PDK4*	*PDPK1*	*PFTK1*	*PFTK2*	*PHKG1*
PHKG2	*PI4K2A*	*PI4K2B*	*PI4KA*	*PI4KB*	*PIK3C2A*	*PIK3C2B*	*PIK3C2G*
PIK3C3	*PIK3CA*	*PIK3CB*	*PIK3CD*	*PIK3CG*	*PIK3R1*	*PIK3R2*	*PIK3R3*
PIK3R4	*PIK3R5*	*PIK3R6*	*PIKFYVE*	*PIM1*	*PIM2*	*PIM3*	*PINK1*
PIP4K2A	*PIP4K2B*	*PIP4K2C*	*PIP5K1A*	*PIP5K1B*	*PIP5K1C*	*PIP5KL1*	*PIPSL*
PKLR	*PKMYT1*	*PKN1*	*PKN2*	*PKN3*	*PLK1*	*PLK2*	*PLK3*
PLK4	*PNCK*	*PRAGMIN*	*PRKAA1*	*PRKAA2*	*PRKACA*	*PRKACB*	*PRKACG*
PRKCA	*PRKCB*	*PRKCD*	*PRKCE*	*PRKCG*	*PRKCH*	*PRKCI*	*PRKCQ*
PRKCZ	*PRKD1*	*PRKD2*	*PRKD3*	*PRKDC*	*PRKG1*	*PRKG2*	*PRKX*
PRKY	*PRPF4B*	*PSKH1*	*PSKH2*	*PTK2*	*PTK2B*	*PTK6*	*PTK7*
PXK	*RAC1*	*RAF1*	*RAGE*	*RET*	*RIOK1*	*RIOK2*	*RIOK3*
RIPK1	*RIPK2*	*RIPK3*	*RIPK4*	*RNASEL*	*ROCK1*	*ROCK2*	*ROR1*
ROR2	*ROS1*	*RPS6KA1*	*RPS6KA2*	*RPS6KA3*	*RPS6KA4*	*RPS6KA5*	*RPS6KA6*
RPS6KB1	*RPS6KB2*	*RPS6KC1*	*RPS6KL1*	*RYK*	*SBK1*	*SBK2*	*SCYL1*
SCYL2	*SCYL3*	*SGK1*	*SGK196*	*SGK2*	*SGK269*	*SGK3*	*SGK493*
SIK1	*SIK2*	*SIK3*	*SLK*	*SMG1*	*SNRK*	*SPEG*	*SPHK1*
SPHK2	*SRC*	*SRM*	*SRMS*	*SRPK1*	*SRPK2*	*SRPK3*	*STK10*
STK11	*STK16*	*STK17A*	*STK17B*	*STK19*	*STK24*	*STK25*	*STK3*
STK31	*STK32A*	*STK32B*	*STK32C*	*STK33*	*STK35*	*STK36*	*STK38*
STK38L	*STK39*	*STK4*	*STK40*	*STRADA*	*STRADB*	*STYK1*	*SYK*
TAF1	*TAF1L*	*TAOK1*	*TAOK2*	*TAOK3*	*TBCK*	*TBK1*	*TEC*
TEK	*TESK1*	*TESK2*	*TEX14*	*TGFBR1*	*TGFBR2*	*TIE1*	*TLK1*
TLK2	*TNIK*	*TNK1*	*TNK2*	*TNNI3K*	*TP53RK*	*TRIB1*	*TRIB2*
TRIB3	*TRIM24*	*TRIM28*	*TRIM33*	*TRIO*	*TRPM6*	*TRPM7*	*TRRAP*
TSSK1B	*TSSK2*	*TSSK3*	*TSSK4*	*TSSK6*	*TTBK1*	*TTBK2*	*TTK*
TTN	*TXK*	*TYK2*	*TYRO3*	*UHMK1*	*ULK1*	*ULK2*	*ULK3*
ULK4	*VRK1*	*VRK2*	*VRK3*	*WEE1*	*WEE2*	*WNK1*	*WNK2*
WNK3	*WNK4*	*YES1*	*YSK4*	*ZAK*	*ZAP70*		
Cancer-related genes other than kinase genes (n =46)							
APC	*BRCA1*	*BRCA2*	*CCND1*	*CCND2*	*CCND3*	*CDC6*	*CDH1*
CDKN2A	*CDKN2B*	*CHD3*	*COL1A1*	*CTNNB1*	*ESR1*	*ESR2*	*FBXW7*
GAB1	*GATA3*	*HAUS3*	*HRAS*	*IDH1*	*IDH2*	*INPP4A*	*IRS2*
IRS4	*KIAA1468*	*KLHL4*	*KRAS*	*MLH1*	*MYC*	*NF1*	*NF2*
NFKB1	*NFKBIA*	*NFKBIE*	*NRAS*	*PALB2*	*PTEN*	*RB1*	*RHEB*
RNF220	*SNX4*	*SP1*	*TERT*	*TP53*	*USP28*		

© 2020 by the authors. Licensee MDPI, Basel, Switzerland. This article is an open access article distributed under the terms and conditions of the Creative Commons Attribution (CC BY) license (http://creativecommons.org/licenses/by/4.0/).
